# Determinants of health insurance ownership among women in Kenya: evidence from the 2008–09 Kenya demographic and health survey

**DOI:** 10.1186/1475-9276-13-27

**Published:** 2014-03-31

**Authors:** James K Kimani, Remare Ettarh, Charlotte Warren, Ben Bellows

**Affiliations:** 1 Population Council, General Accident Insurance House, Ralph Bunche Road, P.O. Box 17643–00500, Nairobi, Kenya; 2 Faculty of Medicine, University of British Columbia, 2775 Laurel Street, Vancouver, British Columbia V5Z 1 M9, Canada; 3 Population Council, 4301 Connecticut Avenue NW, Suite 280, Washington, DC 20008, USA

**Keywords:** Social health insurance, National Hospital Insurance Fund, Women, Kenya

## Abstract

**Background:**

The Government of Kenya is making plans to implement a social health insurance program by transforming the National Hospital Insurance Fund (NHIF) into a universal health coverage program. The objective of this study was to examine the determinants associated with health insurance ownership among women in Kenya.

**Methods:**

Data came from the 2008–09 Kenya Demographic and Health Survey, a nationally representative survey. The sample comprised 8,435 women aged 15–49 years. Descriptive statistics and multivariable logistic regression analysis were used to describe the characteristics of the sample and to identify factors associated with health insurance ownership.

**Results:**

Being employed in the formal sector, being married, exposure to the mass media, having secondary education or higher, residing in households in the middle or rich wealth index categories and residing in a female-headed household were associated with having health insurance. However, region of residence was associated with a lower likelihood of having insurance coverage. Women residing in Central (OR = 0.4; p < 0.01) and North Eastern (OR = 0.1; p < 0.5) provinces were less likely to be insured compared to their counterparts in Nairobi province.

**Conclusions:**

As the Kenyan government transforms the NHIF into a universal health program, it is important to implement a program that will increase equity and access to health care services among the poor and vulnerable groups.

## Background

Social health protection systems are mechanisms that countries use to address the challenges related to providing access to health care services to their citizens, especially the poor segments of the population. The benefits of extending social protection in health include reducing financial barriers associated with access to health care services and protection from financial catastrophe and impoverishment related to health care expenditures [1-5]. One of the categories of social health protection systems is the social health insurance, which is a financing scheme where monies are pooled into a common fund and used for paying for healthcare costs of members. Contributions are usually collected from workers, self-employed individuals, businesses and in some cases the government, particularly where a universal coverage model is adopted [2,5]. Generally, but not always, contributions have ensured that the rich contribute more than the poor but contributions do not typically vary with health status [6].

In Kenya, a universal social health insurance scheme has not been implemented; however, in November 2004, the government introduced the National Social Health Insurance Fund (NSHIF) Bill in parliament. The Bill was passed by parliament in December 2004 [7], but the President declined to assent to the Bill and sent it back to parliament due to a number of concerns. One of the concerns was that the Bill was deemed too expensive to implement and financially unsustainable [8]. As the government prepares to re-introduce the NSHIF legislation in parliament, it is important to have a better understanding of factors associated with participation in the current National Hospital Insurance Fund (NHIF), particularly among the poor, as well as a determination of the proportion of individuals without access to health insurance among this demographic group. The NSHIF will build on the existing NHIF framework and, therefore, such evidence is imperative in order to implement an effective NSHIF. It is hoped that the proposed NSHIF will have mechanisms that will increase equity and access to health care services by all population groups.

Literature focusing on the determinants of participation in health insurance schemes in Kenya and Africa in general is limited. Studies conducted in a number of sub-Saharan African countries showed that employment in the formal sector was significantly associated with access to health insurance relative to being employed in the informal sector [2,3,9]. The low participation of individuals in the informal sector was attributed to a number of factors, including low and non-regular incomes, insecure employment, and insurance scheme design features (e.g., inflexible payment schedules and lack of awareness about insurance schemes) that are not adapted to people’s needs and preferences. In Kenya, it is estimated that 31.6% and 26.3% of the total workforce are engaged in the informal and formal sectors, respectively, while 42.1% are engaged in small-scale farming and pastoralist activities [10]. Other factors that have been cited by previous research as predictors of health insurance ownership include income, education, household wealth status, marital status, age, place of residence [11-20].

In Kenya, more than four out of 10 (46.6%) individuals live below the poverty line [21]. Data from the national health accounts show that more than a third of the poor who were ill did not seek care compared to only 15% of the rich [22]. Additionally, according to the 2005/06 national health accounts, 36% of funds to the health sector came from households and out of these, the out-of-pocket expenditure accounted for more than 29% [23]. These findings raise concern about equity and financial accessibility to health care by a majority of people in Kenya, particularly the poor who are highly vulnerable to economic shocks that result from catastrophic out-of-pocket health expenditure. Existing studies show that the poor are more likely to get sick, less likely to use preventive and curative health care, and consequently, have higher mortality rates. According to these studies, one of the factors responsible for these challenges is high out-of-pocket payments for health care [24-26]. The 2010 World Health Report and the 2010 Millennium Development Goals report underscore the importance of reducing disparities in access to health care, particularly among the poor and marginalized groups through universal health coverage [27,28]. Extending access to health care to all segments of the population, including the poor is an important objective of the Kenyan government’s national health sector strategic plan and national development agenda as outlined in the Kenya Vision 2030 policy framework [29-31].

Besides the NHIF, in Kenya, individuals can access health insurance through private insurance firms and to some extent community-based health insurance (CBHI) organizations. Due to cost considerations, private health insurance is predominantly accessible to the middle and higher-income groups [9]. Community-based health insurance is relatively new in Kenya having been established in 1999, and, as a result it has limited coverage [3] According to the Kenya Community-Based Health Financing Association (KCBHFA), currently, there are nine institutions offering community health financing schemes with 410,997 beneficiaries or about 1% of the population covered [32]. In Africa, countries such as Burkina Faso, Senegal, Tanzania and Ghana have well developed CBHI schemes that are recognized by the national governments as a key component in the national health financing strategy [33-39]. Findings from these studies suggest that CBHI schemes have the ability to reach marginalized population groups such as the poor, women and children, however, more support and strategies from governments are needed to enhance their development and sustainability. Existing evidence shows that in sub-Saharan Africa there are various types of approaches that have been used to ensure expansion of health insurance coverage to the population. A review conducted by the World Bank on the impact of universal coverage schemes in developing countries showed that Rwanda and Nigeria are examples of countries with more than one insurance scheme targeting different population segments with aim of working toward universal health coverage [40]. In Namibia and South Africa they have voluntary insurance mechanisms that include private health insurance [41].

The NSHIF legislation seeks to transform the current National Hospital Insurance Fund (NHIF) into a universal health coverage program, which will ensure equity and access to healthcare services by all citizens. One of the criticisms of the NHIF is its failure to reach out to the majority of Kenyans, especially the poor and those in the informal sector [2,3,9,42]. While the NHIF has a component for people in the informal sector, however, some of the design features of the program act as critical barriers. For example, the NHIF imposes a penalty that is five times the contribution amount for those who do not make their payments by the due date. This regulation particularly hurts the poor, the unemployed and casual workers in the informal sector, who do not have a steady income that would enable them, pay their contributions regularly. As the government of Kenya makes plans to transform the National Hospital Insurance Fund (NHIF) into a universal health coverage program, it is imperative to examine what factors are associated with health insurance ownership in Kenya, particularly among vulnerable sub-groups in the population. The aim of this study was to examine the determinants associated with health insurance ownership among women in Kenya.

## Methods

### Study design and sampling

Data came from the 2008–09 Kenya Demographic and Health Survey (KDHS), a nationally representative survey [43]. The sampling frame included a total of 400 primary sampling units across the eight provinces. Multi-stage cluster sampling was used to select 8,444 women aged 15 to 49 years across the eight provinces of Kenya with stratification for rural and urban residence.

### Measures

The outcome variable was whether a woman was covered by any health insurance (*Yes or No*). The explanatory variables examined in the study were selected based on factors cited in the literature as influencing health insurance ownership and included respondents occupation groups (categorized into three employment categories – *formal, informal and not working*); marital status, categorized into *never married, married and formerly married*; exposure to the mass media (grouped into frequency of reading newspaper, listening to radio and watching television), education level, grouped into *no formal education, primary education, secondary education or higher;* age of woman in years, grouped into *15*–*19, 20–24, 25–29, 30–34, 35–39, 40–44 and 45–49;* gender of household head (*male or female*); number of household members, grouped into *1*–*4 members and 5 or more members* (the average number of household members was 5 and so the variable was categorized as below 5 or 5 and above)*;* household wealth status categorized into *poorest/poorer*, *middle and richer/richest*; place of residence (*urban or rural*); and geographical province of residence (*Central, Coast, Eastern, North Eastern, Nairobi, Nyanza, Rift Valley and Western*).

### Data analysis

For this paper, a total of 8,435 women with complete data on the key outcome variable were included in the analyses. Descriptive statistics and multivariate logistic regression analysis were used to describe the characteristics of the sample and to identify factors associated with health insurance ownership. For the bivariate analysis, Pearson’s chi-square test (*X*^2^) was used to test the association between health insurance ownership and the explanatory variables. Data analysis was performed using STATA® version 10 and statistical adjustments were made to get robust standard errors since the sampling of respondents in the KDHS involved stratification and clustering [44,45].

### Ethical considerations

The study involved secondary analysis of data from the KDHS which excluded participant identifiers. The survey protocol was approved by the Scientific and Ethical Review Committee of Kenya Medical Research Institute (KEMRI).

## Results

### Descriptive analysis

Table 1 presents the results from the descriptive analysis. Only 7% of the women had health insurance and among these, a higher proportion were covered by employer-based health insurance (4%), while less than 1% were covered by community-based health insurance schemes (results not shown). Many of the women were unemployed while 30% and 25% were employed in the informal and formal sectors, respectively. The majority of the women were married, listened to radio, had primary level of education, lived in male-headed households and resided in rural areas.

**Table 1:** Health insurance ownership and socio-demographic characteristics of study population

**Variable**	**N**	**%**
**Covered by health insurance**		
No	7,831	92.8
Yes	604	7.2
**Employment sector**		
Formal employment	2,116	25.1
Informal employment	2,568	30.5
Not working	3,739	44.4
**Marital status**		
Never married	2,540	30.1
Married	5,041	59.7
Formerly married	863	10.2
**Exposure to media**		
**Frequency of reading newspaper**		
Not all	4,921	58.3
Sometimes	2,949	35.0
Almost everyday	566	6.7
**Frequency of listening to radio**		
Not all	1,551	18.4
Sometimes	2,006	23.8
Almost everyday	4,881	57.8
**Frequency of watching television**		
Not all	4,602	54.5
Sometimes	1,636	19.4
Almost everyday	2,204	26.1
**Education**		
No education	1,242	14.7
Primary	4,404	52.2
Secondary or higher	2,798	33.1
**Age (Years)**		
15-19	1,767	20.9
20-24	1,744	20.7
25-29	1,423	16.9
30-34	1,180	14.0
35-39	930	11.0
40-44	730	8.7
45-49	670	7.9
**Gender of household head**		
Male	5,352	63.4
Female	3,092	36.6
**Number of household members**		
1-4 members	3,363	39.8
5+ members	5,081	60.2
**Household wealth status**		
Poorest/poorer	2,983	35.3
Middle	1,455	17.2
Richer/richest	4,006	47.4
**Place of residence**		
Urban	2,615	31.0
Rural	5,829	69.0
**Province**		
Central	973	11.5
Coast	1,149	13.6
Eastern	1,127	13.4
North Eastern	608	7.2
Nairobi	952	11.3
Nyanza	1,318	15.6
Rift Valley	1,278	15.1
Western	1,039	12.3
**Total**	8,444	100.0

Note: Percentages may not add up to 100 due to rounding off.

The results of the bivariate analysis of the association between health insurance ownership and explanatory variables are shown in Table 2. A significantly higher proportion of women with health insurance were employed in the formal sector (17%) while 4% were employed in the informal sector and a similar proportion were unemployed (p < 0.001). Having health insurance was significantly associated with being married (8%), listening to radio almost every day (58%), reading newspaper almost every day (35%) and watching television almost every day (19%), having secondary school education and higher (18%), belonging to wealthier households (14%) and residing in urban areas (15%).

**Table 2:** Bivariate analysis for associations between health insurance ownership and explanatory variables

	**Covered by health insurance**		
**Variable**	**Total number**	**N (%)**	***p-values***
**Employment sector**			
Formal employment	2,114	353 (16.7)	***
Informal employment	2,565	102 (4.0)	
Not working	3,735	147 (3.9)	
**Marital status**			
Never married	2,538	158 (6.2)	***
Married	5,035	411 (8.2)	
Formerly married	862	35 (4.1)	
**Education**			
No education	1,239	6 (0.5)	***
Primary	4,400	100 (2.3)	
Secondary or higher	2,796	498 (17.8)	
**Exposure to media**			
**Frequency of reading newspaper**			
Not all	4,913	1.9	***
Sometimes	2,948	10.6	
Almost everyday	566	35.0	
**Frequency of listening to radio**			
Not all	1,551	18.4	***
Sometimes	2,004	23.8	
Almost everyday	4,877	57.8	
**Frequency of watching television**			
Not all	4,595	1.7	***
Sometimes	1,636	6.2	
Almost everyday	2,202	19.3	
**Age (Years)**			
15-19	1,765	53 (3.0)	***
20-24	1,742	86 (4.9)	
25-29	1,422	123 (8.7)	
30-34	1,178	118 (10.0)	
35-39	930	91 (9.8)	
40-44	729	72 (9.9)	
45-49	669	61 (9.1)	
**Gender of household head**			
Male	5,345	393 (7.4)	0.368
Female	3,090	211 (6.8)	
**Number of household members**			
1-4 members	3,360	305 (9.1)	***
5+ members	5,075	299 (5.9)	
**Household wealth status**			
Poorest/poorer	2,977	18 (0.6)	***
Middle	1,455	44 (3.0)	
Richer/richest	4,003	542 (13.5)	
**Place of residence**			
Urban	2,611	395 (15.1)	***
Rural	5,824	209 (3.6)	
**Province**			
Central	972	40 (4.1)	***
Coast	1,149	67 (5.8)	
Eastern	1,127	62 (5.5)	
North Eastern	606	1 (0.2)	
Nairobi	951	230 (24.2)	
Nyanza	1,316	82 (6.2)	
Rift Valley	1,276	84 (6.6)	
Western	1,038	38 (3.7)	
Total	8,444	604 (7.2)	

*p < 0.05; **p < 0.01; ***p < 0.001; *X*^*2*^ was used to test the association between health insurance ownership and explanatory variables.

### Multivariate analysis

The results of the multivariate logistic regression analysis for determinants of health insurance coverage are shown in Table 3. Being employed in the formal sector was significantly associated with a higher probability of having health insurance compared to being unemployed (OR = 2.2; p < 0.001). Married women were significantly associated with having health insurance compared to never married women (OR = 1.8; p < 0.05). Exposure to the mass media was significantly associated with health insurance ownership. Specifically, women who read newspapers, listened to radio or watched television sometimes or almost every day had a higher probability of having health insurance compared to those who never did. Education was a significant predictor of having insurance coverage. Women who had attained primary level of education (OR = 4.4; p < 0.01) and secondary education or higher (OR = 10.9; p < 0.001) were associated with a higher likelihood of having health insurance compared to those with no formal education. Generally, controlling for all other variables, the probability of having health insurance tended to increase with age although non-significant results were observed for age categories 20–24 years and 40–44 years. Other significant determinants of having health insurance were the gender of household head and household wealth status. Women living in female-headed households were significantly more likely to be insured (OR = 1.7; p < 0.01) compared to their counterparts in male-headed households. The probability of having health insurance increased as the level of household wealth index increased. Women from wealthier households were six times more likely to have health insurance coverage compared to those from poor households. Women residing in the geographic provinces of Central and North Eastern had a significantly lower likelihood of having health insurance compared to their counterparts in Nairobi province.

**Table 3:** **Adjusted odds ratios (ORs) and 95**% **confidence intervals (CIs) for determinants of health insurance ownership**

**Variable**	**Adjusted OR**	**95% CI**
**Employment sector *(Ref = Not working)***		
Formal employment	2.2***	[1.5-3.2]
Informal employment	1.5	[0.8-3.1]
**Marital status *(Ref = Never married)***		
Married	1.8*	[1.3-2.5]
Formerly married	0.5	[0.2-1.1]
**Exposure to media**		
**Frequency of reading newspaper *(Ref = Not at all)***		
Sometimes	1.6*	[1.0-2.6]
Almost everyday	3.5***	[2.0-6.0]
**Frequency of listening to radio *(Ref = Not at all)***		
Sometimes	1.5	[0.9-2.5]
Almost everyday	1.7*	[1.0-3.1]
**Frequency of watching television *(Ref = Not at all)***		
Sometimes	2.3***	[1.4-3.5]
Almost everyday	2.6***	[1.7-4.2]
**Education *(Ref = No education)***		
Primary	4.4**	[1.6-12.2]
Secondary or higher	10.9***	[3.6-33.1]
**Age (Years) *(Ref = 15–19)***		
20-24	1.6	[0.8-3.5]
25-29	2.5**	[1.3-4.9]
30-34	2.0*	[1.2-3.5]
35-39	2.6**	[1.4-4.8]
40-44	1.6	[0.9-2.9]
45-49	4.0***	[2.0-7.8]
**Gender of household head *(Ref = Male)***		
Female	1.7**	[1.2-2.3]
**Number of household members *(Ref = 1–4)***		
5+ members	1.0	[1.0-1.1]
**Household wealth status *(Ref = Poorest/poorer)***		
Middle	3.2**	[1.5-6.9]
Richer/richest	6.3**	[3.0-13.2]
**Place of residence *(Ref = Urban)***		
Rural	1.0	[0.6-1.4]
**Province *(Ref = Nairobi)***		
Central	0.4**	[0.2-0.8]
Coast	0.8	[0.4-1.6]
Eastern	1.0	[0.5-1.8]
North Eastern	0.1*	[0.0-0.7]
Nyanza	1.1	[0.6-1.9]
Rift Valley	1.4	[0.9-2.2]
Western	0.6	[0.3-1.3]

*p < 0.05; **p < 0.01; ***p < 0.001.

## Discussion

The objective of this paper was to examine the determinants of health insurance ownership among women in Kenya. The findings showed that a high proportion of women (93%) have no access to any type of health insurance. Our findings also showed that more women in the formal sector than informal sector had been insured. After controlling for all other variables, being employed in the formal sector was still associated with having health insurance. This finding corroborates evidence from previous studies, which demonstrated that employment in the formal sector is an important determinant of being insured [2,3,9]. The differences in insurance coverage between the formal and informal sectors have important implications on the proposed plans to establish a social health insurance program in Kenya. One objective of comprehensive social health insurance is to ensure that all population groups irrespective of their socio-economic status have access to quality and affordable health care. Our findings suggest that more efforts are needed to increase health insurance coverage of individuals in the informal sector. Previous research has shown that unlike in the formal sector, it is difficult to assess incomes and collect income taxes from workers employed in the informal sector [9] and, as a consequence, deduction of contributions for the proposed social health insurance program can be a challenge. This means that lack of suitable mechanisms for collecting contributions from employees in the informal sector could hamper the implementation and sustainability of the proposed social health insurance program. However, evidence shows that many workers in the informal sector participate in microfinance institutions such as savings and credit cooperative organizations (SACCOs) and community-based groups (e.g., merry-go-rounds) [46] and, therefore, these organized units can be important platforms through which contributions are collected and submitted to the social health insurance program.

Our study findings also showed that a number of factors are significant determinants of health insurance ownership including marital status (specifically, being married), education, age, gender of household head and household wealth status. However, geographical region was associated with a lower probability of having health insurance. Similar to previous research [11,12], our findings showed that being married was associated with having health insurance coverage compared to never been married and formerly married. This suggests that having a spouse/partner is beneficial possibly because of the financial support derived from being in a dual-income household, which translates into more opportunities for accessing health insurance coverage. Another plausible reason is that a spouse/partner can be insured through the other’s insurance coverage from the employer. Exposure to the media through reading newspapers, listening to radio or watching television was associated with having health insurance. Education was also an important determinant of having insurance coverage. More educated women were more likely to have health insurance relative to women with no formal education. This finding corroborates evidence from previous studies which demonstrated that education is an important predictor of having health insurance [11,47,48]. Consistent with previous studies [14,17,12], our findings demonstrated that the likelihood of health insurance ownership tends to rise with increase in age. One possible explanation for this outcome is that financial security increases with age, which in turn increases the ability to purchase health insurance policies. Another important predictor of health insurance ownership was the gender of the household head. Women residing in female-headed households were more likely to be insured compared to their counterparts in male-headed households. We could not find a plausible explanation for this observation and future research needs to investigate this outcome. Household wealth status was also an important determinant for health insurance ownership. The likelihood being insured increased as one moved up the household wealth index. This finding is consistent with previous studies which showed that wealthier households had a higher likelihood of being insured [13,19,12]. Region of residence was also a significant predictor of health insurance ownership. Specifically, women residing in the geographical regions of Central, North Eastern and Western had a lower likelihood of having health insurance compared to Nairobi province. The geographical differential in health insurance coverage could be explained by the fact that Nairobi, which is the capital city of Kenya, is entirely urban and has a higher proportion of the population in the highest wealth quintile and higher literacy levels compared with other geographical regions [43].

The findings from our study have important policy implications. First, the large proportion of women without health insurance and the lower likelihood of poor households to have insurance coverage highlight the need by the government to hasten the move towards social health protection by implementing a National Social Health Insurance Fund. This is to guarantee access to quality healthcare services for the poor and vulnerable segments of the population, as well offer protection against catastrophic out-of-pocket health expenditure associated with high medical costs. To ensure that the vulnerable and poor have access to health care under the NSHIF, the government will need to institute targeted subsidies and exemptions aimed at increasing health insurance coverage, particularly for women resident outside Nairobi. Second, our study shows that people employed in the informal sector are less likely to have health insurance. Considering that the informal sector accounts for the highest proportion of Kenya’s total workforce [10], reaching out to this sector is critical for the successful implementation of the social health insurance scheme.

### Limitations

One limitation of our study is that we were unable to assess the association between health status and having health insurance coverage due to the lack of data on respondents health status (for example, presence of illnesses, frequency of illnesses). Previous studies have shown that health status is an important predictor of health insurance coverage [20,36,39]. Also, no data were collected on out-of-pocket payments and health care utilization; therefore, it was not possible to examine the effect of having health insurance on these two outcomes. Another limitation is that the questionnaire did not collect data on the extent of insurance coverage such as type of services covered and, therefore, we were not able to assess the association between the extent of insurance coverage and health insurance ownership.

## Conclusions

Addressing disparities in access to care among the poor and marginalized demographic groups is a key agenda in the global health debate because it’s a critical factor in accelerating the achievement of the Millennium Development Goals (MDGs). Our study has highlighted important issues that will inform the efforts aimed at establishing a social health insurance program by transforming the National Hospital Insurance Fund (NHIF) into a universal health coverage program. The large proportion of women without health insurance and the lower likelihood of poor households to have insurance coverage underscore the need for a social health insurance program to ensure equitable access to health care. Also, there is need to design and implement targeted initiatives that will increase health insurance coverage among people working in the informal sector. As the Government of Kenya moves toward transforming the NHIF into a universal health program, it is important to implement a program that will increase equity and access to health care services among the poor and vulnerable groups.

## Competing interests

The authors declare that they have no competing interests.

## Authors’ contributions

JK conceptualized the study, conducted the data analyses, participated in the literature review, and prepared the first draft of the manuscript. RE made substantive contribution that informed the data analyses and reviewed the manuscript. CW and BB were involved in revising the manuscript for intellectual content and interpretation of data. All authors are aware that the manuscript is being submitted to the journal. All authors read and approved the final manuscript.
